# Functional balance at rest of hemispheric homologs assessed via normalized compression distance

**DOI:** 10.3389/fnins.2023.1261701

**Published:** 2024-01-25

**Authors:** Annalisa Pascarella, Vittoria Bruni, Karolina Armonaite, Camillo Porcaro, Livio Conti, Federico Cecconi, Luca Paulon, Domenico Vitulano, Franca Tecchio

**Affiliations:** ^1^Istituto per le Applicazioni del Calcolo ‘Mauro Picone’, National Research Council of Italy, Rome, Italy; ^2^Department of Basic and Applied Science for Engineering (SBAI), University of Rome ‘Sapienza’, Rome, Italy; ^3^Faculty of Engineering, Uninettuno University, Rome, Italy; ^4^Department of Neuroscience and Padova Neuroscience Center, University of Padua, Padua, Italy; ^5^Laboratory of Electrophysiology for Translational neuroScience and Laboratory for Agent Based Social Simulation, Institute of Cognitive Sciences and Technologies, National Research Council of Italy, Rome, Italy; ^6^Centre for Human Brain Health and School of Psychology, University of Birmingham, Birmingham, United Kingdom; ^7^Luca Paulon, Independent Researcher, Rome, Italy

**Keywords:** temporal course of the neuronal electrical activity, functional source separation, resting state, neurodynamics, normalized compression distance

## Abstract

**Introduction:**

The formation and functioning of neural networks hinge critically on the balance between structurally homologous areas in the hemispheres. This balance, reflecting their physiological relationship, is fundamental for learning processes. In our study, we explore this functional homology in the resting state, employing a complexity measure that accounts for the temporal patterns in neurodynamics.

**Methods:**

We used Normalized Compression Distance (NCD) to assess the similarity over time, neurodynamics, of the somatosensory areas associated with hand perception (S1). This assessment was conducted using magnetoencephalography (MEG) in conjunction with Functional Source Separation (FSS). Our primary hypothesis posited that neurodynamic similarity would be more pronounced within individual subjects than across different individuals. Additionally, we investigated whether this similarity is influenced by hemisphere or age at a population level.

**Results:**

Our findings validate the hypothesis, indicating that NCD is a robust tool for capturing balanced functional homology between hemispheric regions. Notably, we observed a higher degree of neurodynamic similarity in the population within the left hemisphere compared to the right. Also, we found that intra-subject functional homology displayed greater variability in older individuals than in younger ones.

**Discussion:**

Our approach could be instrumental in investigating chronic neurological conditions marked by imbalances in brain activity, such as depression, addiction, fatigue, and epilepsy. It holds potential for aiding in the development of new therapeutic strategies tailored to these complex conditions, though further research is needed to fully realize this potential.

## 1 Introduction

The effective functioning of a healthy brain hinges on a dynamic balance between the homologous regions of both hemispheres. This balance is facilitated by inter-hemispheric inhibition, a key aspect of brain organization. Essentially, excitatory projections from one hemisphere activate the inhibitory networks of its counterpart, contributing to the formation of surrounding lateral networks (Zatorre et al., 2012; Carson, 2020). The formation of these networks that implement the ‘center on–surround off’ mechanism plays a crucial role in acquiring new functionalities at the neuronal cortical level. It supports the development of motor control (Mahan and Georgopoulos, 2013; Georgopoulos and Carpenter, 2015) and enhances sensory-perceptual acuity (Kolasinski et al., 2017; Grujic et al., 2022). Consequently, the interaction between homologous hemispheric areas regulates the inhibition–excitation balance in networks that control body segments, vital for adaptive plasticity and learning processes (Das and Gilbert, 1999; Graziadio et al., 2010). The inter-hemispheric balance is crucial in chronic conditions such as fatigue (Cogliati Dezza et al., 2015; Ondobaka et al., 2022), and it influences the severity of stroke (Deco and Corbetta, 2011; Pellegrino et al., 2012; Zappasodi et al., 2014; Soleimani et al., 2023) and aging (Cottone et al., 2013). In particular, neuromodulation interventions aimed at relieving fatigue have been observed to restore the physiological homology of primary motor areas (Porcaro et al., 2019) and of the cortico-spinal tracts (Bertoli et al., 2023).

By examining the balance between homologous somatosensory regions in the hemispheres during rest, we can gain insights into the activation capabilities of networks associated with function recovery (Graziadio et al., 2012; Pellegrino et al., 2012), development (Graziadio et al., 2010) and aging (Cottone et al., 2013). In particular, the resting state of neuronal networks (Porcaro et al., 2019) is crucial for understanding chronic alterations such as fatigue, which affects not just specific tasks but the entire individual experience (Spetsieris et al., 2015).

Our study introduces a novel measure to evaluate hemispheric homology through resting neurodynamics. We hypothesize that neurodynamic similarity is greater between homologous areas in the two hemispheres of a single individual than among different individuals. To quantify this similarity, we employ the normalized compression distance (NCD), a parameter-free, quasi-universal measure derived from compressed data file lengths (Cilibrasi and Vitányi, 2005). NCD has proven effective in various applications, such as genome comparison (Nykter et al., 2008), neuronal network behavior (Gomez, 2009), language clustering (Cilibrasi and Vitányi, 2005; Yoshizawa et al., 2010), and music analysis (Cataltepe et al., 2007; Bello, 2011). Unlike linguistic applications that focus on written texts, musical processing relies on temporal sequences with specific structures (Bello, 2011). Robustness of NCD (Pascarella et al., 2022) makes it ideal for comparing activities in different brain areas, even when recordings are not synchronous. This feature is particularly useful for longitudinal studies examining the effects of aging or disease. Additionally, NCD can compare signals of varying lengths, making it valuable in cases where artifact-induced inconsistencies lead to uneven epoch rejections. Overall, ability of NCD to capture dominant common information in pairwise comparisons positions it as a powerful tool for our research.

## 2 Methods

Twenty-eight healthy, right-handed volunteers participated in the study: 15 males, mean age 51.2 ± 23.5 years, range 24–95; 13 females, mean age 43.2 ± 26.4 years, and range 24–91. The handedness was 83.7 ± 18.2 across subjects, evaluated by the Edinburgh Handedness test. All subjects had normal neurological examinations and did not receive any pharmacological treatment at the time of recording. The Ethical Committee of ‘S. Giovanni Calibita’ Hospital’ approved the study, and subjects signed informed consent forms.

### 2.1 Experimental procedure

Brain magnetic activity in the left and right rolandic regions was collected, while subjects were lying comfortably in a bed with their eyes open and gazing at a central fixation point. A 28-channel magnetoencephalographic (MEG) system (16 internal axial gradiometers and 11 peripheral magnetometers and 1 magnetometer devoted to noise-reduction) centered on C3 and C4 of the international 10–20 electroencephalographic system and covering a total scalp area of approximately 180 cm^2^ was used inside a magnetically shielded room (Vacuumschmelze GMBH). Rest activity was recorded for 3 min in each hemisphere, randomizing the order of left and right acquisition across people. MEG activity was also collected during the electrical stimulation of the contralateral median nerve at the wrist delivered via surface disks (cathode proximal). Elicited electric pulses were 0.2 ms in duration and 631 ms of inter-stimulus interval, with the stimulus intensity set just above the motor threshold inducing a painless thumb twitch, which was visually monitored, with adjustment of the stimulus position if required throughout the stimulation. Left and right median nerves were stimulated separately, totaling approximately 200 artifact-free trials for each. MEG signals were sampled at 1 kHz after proper analogue conditioning (band-pass filtering between 0.48 and 250 Hz) and processed offline. The entire recording procedure lasted about half an hour.

### 2.2 Functional source separation for S1 identification and FS_S1 activity studied at rest

After visual data inspection to exclude trials with saturated signals, we applied the FSS procedure detailed in previous articles (Barbati et al., 2006; Tecchio et al., 2007; Porcaro et al., 2008). FSS can be briefly explained as assuming the recorded data *x* as a linear mix of a set of sources *s* via a mixing matrix *A*. The functional constraint, which identifies the primary somatosensory area devoted to hand perception (FS_S1), quantifies the responsiveness to the median nerve at the latency known to correspond to the stimulus’ arrival in S1 (Appendix 1). In all subjects, we calculated left and right FS_S1 which on average were positioned in the postcentral gyrus wall (Table 1).

**Table 1:** FS_S1 position.

	*X*	*y*	*z*
FS:S1sn	−42 ± 4	−15 ± 7	57 ± 3
FS:S1dx	46 ± 3	−11 ± 8	59 ± 5

Localization results were averaged across subjects after the normalization of individual data in the MNI space (MNI coordinates, mm). The inverse problem was solved using a single equivalent current dipole model in a homogenously conducting sphere, which best fit the head, applied to the MEG distribution of left FS_S1 (FS_S1sn) or right FS_S1 (FS_S1dx) provided by FSS.

**Figure 1:**
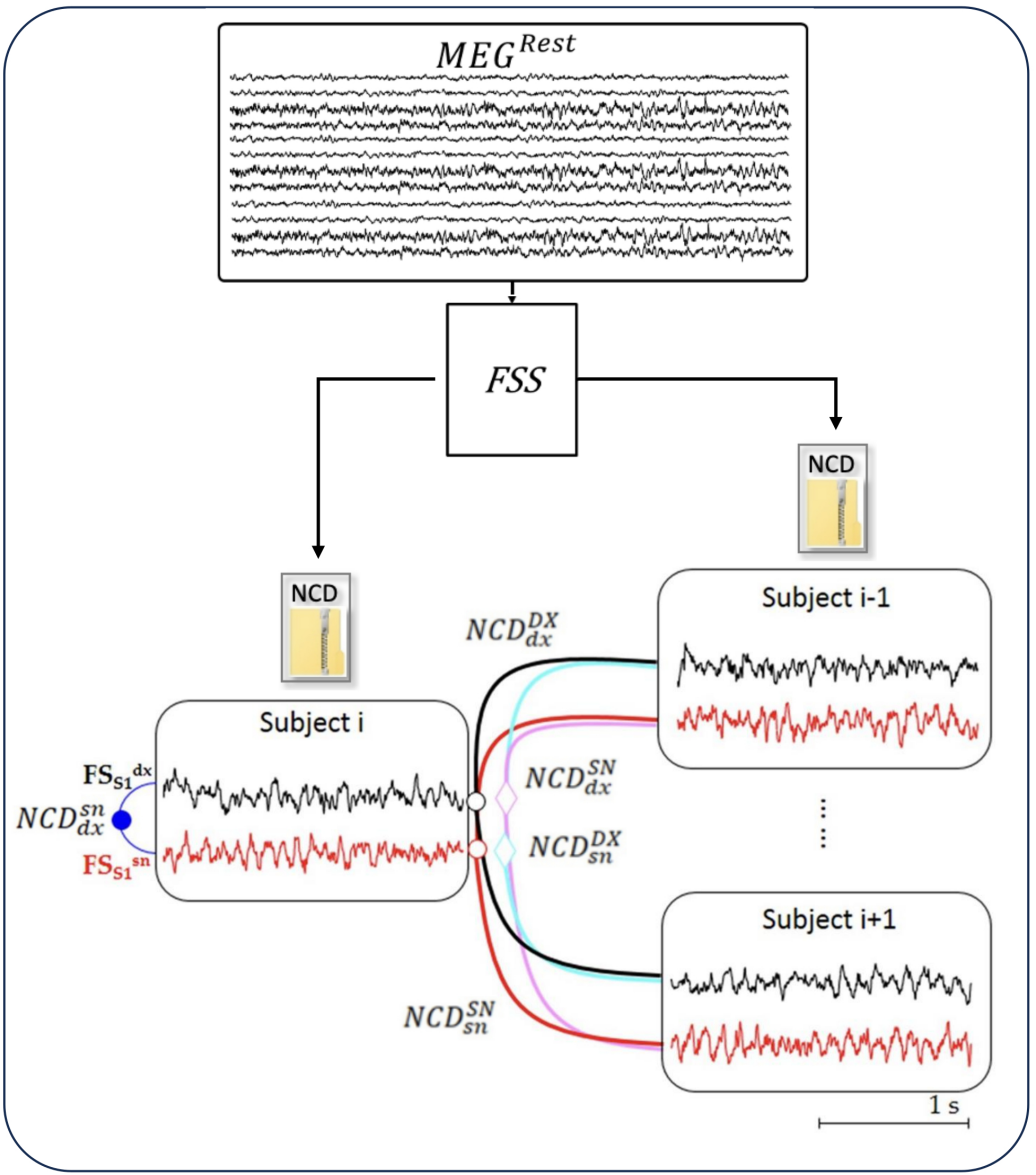
NCD of resting-state neurodynamics. The Functional Source Separation (FSS) algorithm derives from the MEG data the resting-state neurodynamics of S1 sources in the left and right hemispheres ( $FS_{S1}^{sn}\left(t\right)$ and $FS_{S1}^{dx}\left(t\right)$ , respectively) of each subject (i = 1, 28). From time signal of the sources, the normalized compression distance (NCD) calculates the homologous similarities.

A semi-automatic artifact rejection procedure (Barbati et al., 2004) was applied to MEG data, recorded while the subject was at rest, to minimize the contribution of non-cerebral sources (such as the heart, eyes, and muscles), which can critically exceed the brain signal in absence of stimulus-synchronized average noise-reduction. Afterward, we multiplied the artifact-free resting MEG data by the inverse of the demixing matrix of FS_S1 (*W*_FS_S1_ = 1/*A*_FS_S1_) and we obtained FS_S1 activity in the resting state (Figure 1; Appendix 1).

### 2.3 Normalized compression distance

The *Normalized Compression Distance* (NCD; Appendix 2) is a quasi-universal metric, in the sense that it has been defined in order to simultaneously detect *all* similarities between pieces that other effective distances detect separately (Cilibrasi and Vitányi, 2005). In other terms, NCD is based on the concept that two signals are similar if we can significantly “compress” one using the information of the other. As a result, NCD has the potential to minimize every computable similarity distance up to a certain level of error, depending on the quality of the compressor. This means that NCD captures the dominant similarity over all possible features for every pair of objects compared, up to the stated precision.

We must remember that a lossless compressor acts as an invertible mapping function of a signal into a binary sequence. The length of this binary sequence reveals the amount of compression. Hence, the NCD computed between two signals x and y, i.e., NCD(x,y) is defined as

$$NCD\left(x,y\right)=\frac{C\left(xy\right)-\min\left(C\left(x\right),C\left(y\right)\right)}{\max\left(C\left(x\right),C\left(y\right)\right)}\text{,}$$

where C(xy) denotes the compressed size (length of the binary sequence that has been obtained by applying the compressor C) of the concatenation of x and y, wherein C(x) denotes the compressed size of x, and C(y) denotes the compressed size of y. Given the definition, NCD varies between 0 (equal signals) and 1 (signals with no common information). In this study, the compressed size has been measured in terms of number of bits per sample, which is the average number of bits used for coding each sample of the considered signal.

**Figure 2:**
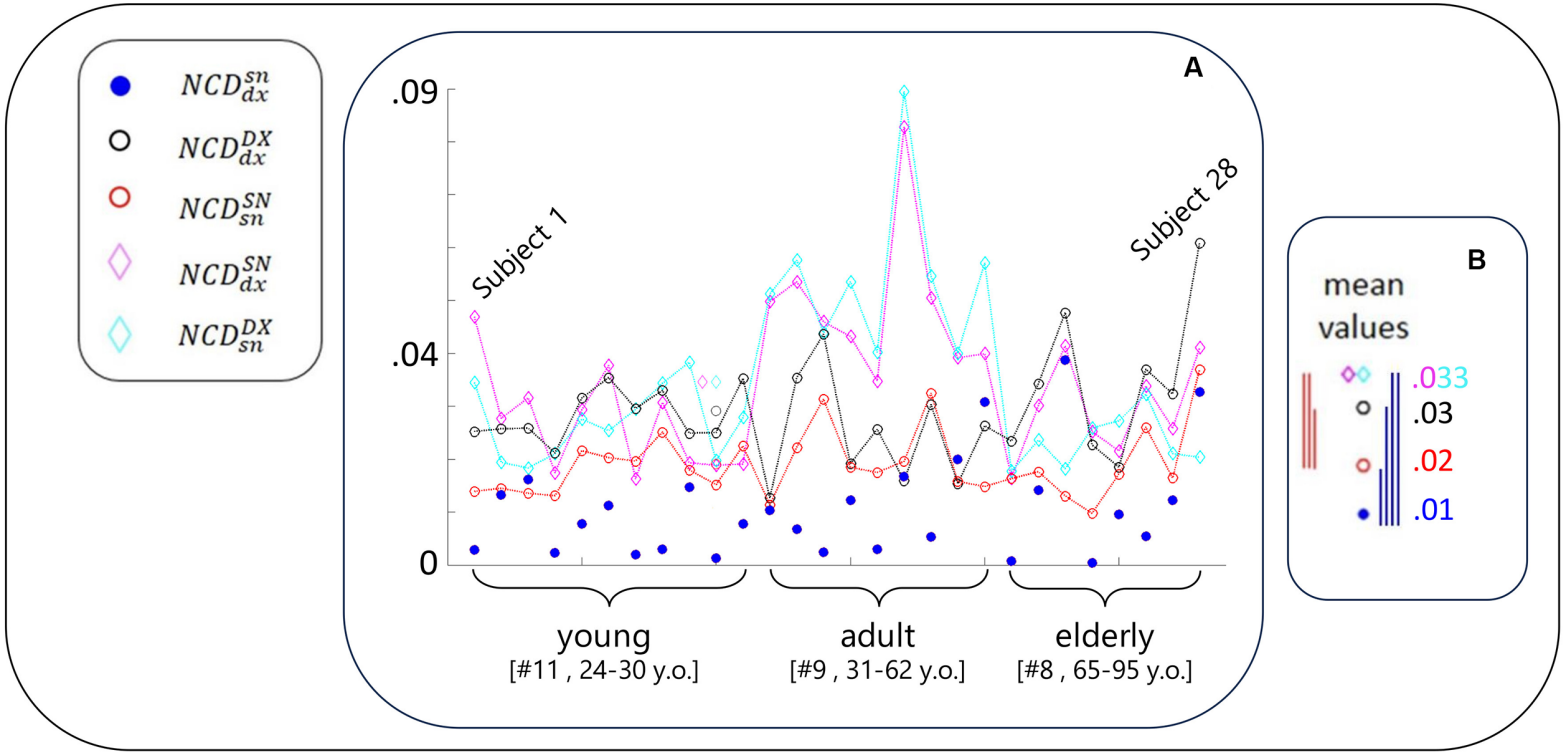
NCD-estimated similarity of local neurodynamics intra- and inter-subjects. **(A)** With the codes included in the legend and introduced in Figure 1 legend, for each subject, NCD between the rest S1 neurodynamics in the left and right hemispheres. Data of the subjects are displayed in order of their age, each equidistant from the successive, range [24–95] years; thus, Subject 1 is the youngest and Subject 28 is the oldest. The greater variability of the blue full circles in the elderly group is visible, including the smaller and biggest values. **(B)** The five mean values of NCD in the same scale of the values of the single subject. The vertical segments express the differences between corresponding values (color code defined by the lower values, that is the most similar neurodynamics. Blue segments express the statistical results that homologous S1 neurodynamics within the same subject were more similar than all other cases (Table 1); red segments report that self-similarity within the left-dominant hemispheres was higher than all other cases (but ${NCD}_{dx}^{sn}$ ; Table 1).

We used the following notation for NCD computed between the resting-state activity of FS_S1 (Figure 2):

-
$NCD_{dx}^{sn}$ : right (dx) and left (sn) of the same person (blue dot)
-
$NCD_{dx}^{DX}$ : one person’s right with any other’s right (DX), considered the mean across the 27 NCD values (black empty circle)
-
$NCD_{sn}^{SN}$ : similar to $NCD_{dx}^{DX}$ for the left FS_S1 (red empty circle)
-
$NCD_{dx}^{SN}$ : similar to $NCD_{dx}^{DX}$ for one person’s right with any other’s left (magenta empty diamond)
-
$NCD_{sn}^{DX}$ : similar to $NCD_{dx}^{DX}$ for one person’s left with any other’s right (cyan empty diamond)

Of note, the mean was considered a good representative as the coefficients of variation were 2% for $NCD_{sn}^{SN}$ and $NCD_{dx}^{DX}$ and 5% for $NCD_{dx}^{SN}$ and $NCD_{sn}^{DX}$ .

In particular, in the notation adopted, the apex and subscript position are irrelevant; $NCD_{dx}^{sn}$ and $NCD_{sn}^{dx}$ are equal. This is consistent with the aforementioned concept of distance.

### 2.4 Study design and statistical analysis

We calculated NCD between the neurodynamics of the hand somatosensory representation (FS_S1) at rest. We tested the working hypothesis that the similarity between the left and right homologous areas in single subjects is greater than across the entire group. Furthermore, we compared the FS_S1 activities in the same hemispheres of different people (every activity, within separately the left and the right hemispheres) to test whether a dominance-related similarity exists.

As the NCD distributions differed from Gaussian by Shapiro–Wilk test, we calculated the non-parametric paired-sample Wilcoxon signed-rank two-tailed for all comparisons. Instead, to evaluate the significance of changes in dependence on age, we applied independent-sample comparisons. The level of significance of the test was measured with the conventional *value of p* (*p* < 0.05). To test for stability of the results, we calculated mean and median.

## 3 Results

### 3.1 The similarity of S1 neurodynamics is greater in intra–than inter-subjects

As is clear from Figure 2, and Tables 2; 3, the similarity between the neurodynamics of right and left S1 areas within a single subject ( $NCD_{dx}^{sn}$ , Table 2, Figure 2, blue full circle) was greater than the four similarities with other subjects: $NCD_{sn}^{SN}$ (Figure 2, red empty circle), $NCD_{dx}^{DX}$ (Figure 2, black empty circle), $NCD_{dx}^{SN}$ (Figure 2, magenta empty diamonds), and $NCD_{sn}^{DX}$ (Figure 2, cyan empty diamonds).

**Table 2:** Intra–and inter-subjects NCD values of resting-state S1 neurodynamics.

	$NCD_{dx}^{sn}$	$NCD_{sn}^{SN}$	$NCD_{dx}^{DX}$	$NCD_{dx}^{SN}$	$NCD_{sn}^{DX}$
Mean	0.011	0.019	0.029	0.035	0.035
Median	0.009	0.018	0.026	0.029	0.033

**Table 3:** Statistical comparison between NCD of resting-state S1 activities intra–and inter-subjects.

	$NCD_{dx}^{DX}$	$NCD_{sn}^{SN}$	$NCD_{dx}^{SN}$	$NCD_{sn}^{DX}$
$NCD_{dx}^{sn}$	2 10^−7^	5 10^−5^	3 10^−8^	4·10^−8^
$NCD_{sn}^{SN}$	6 10^−5^		1 10^−7^	
$NCD_{dx}^{DX}$			0.15	

The table reports the *p*-values of the Wilcoxon rank-sum test by comparing 28 values for the conditions listed in the first column with the 28 values for other columns. For each row, all comparisons indicated smaller values for the condition listed in the first column ( ${NCD}_{dx}^{sn}$ , ${NCD}_{sn}^{SN}$ showed higher similarity, see Figure 2; Table 2).

### 3.2 Hand-related hemispheric dominance

As readable from Figure 2 and Table 2 and statistically evaluated (Table 3), the similarity between the individual neurodynamics and all other subjects in the left hemisphere ( $NCD_{sn}^{SN}$ ) was greater than in the other three comparisons: $NCD_{dx}^{DX}$ , $NCD_{dx}^{SN}$ , $NCD_{sn}^{DX}$ .

### 3.3 Inter-subject similarity dependence on age

We observed that the similarity between individual left and right FS_S1 neurodynamics became increasingly variable with age (Figure 2, blue circle). By subdividing the entire group into three age-dependent classes (11 young people between 24 and 30 years old, 9 adult people between 32 and 61 years old, and 8 elderly people between 65 and 95), the coefficients of variance were 0.75, 0.78, and 1. In agreement, we can observe that the elderly group includes the smallest (subjects 21 and 24) and biggest (subjects 23 and 28) $NCD_{sn}^{dx}$ values. Given this observation, we applied the Fisher test to compare the variances across the groups and found that the elderly group has a higher variance than the young (*p* = 0.009).

## 4 Discussion

In this study, we utilized a novel compression approach to reliably assess the similarity of ongoing electrical activity in the resting state, serving as an indicator of the balance between homologous cortical regions.

The exploration of neurodynamics has significantly advanced our understanding of spontaneous neuronal electrical activity, which results from the interplay of projections to and from a cortical region or network. Previously, ‘morphology similarity’ was used to identify sensory-specific recruitment patterns (Tecchio et al., 2000, 2005), uncovering crucial phenomena in stroke recovery (Tecchio et al., 2000, 2006; Oliviero et al., 2004), effects in individuals with multiple sclerosis (Dell’Acqua et al., 2010), and intra-surgical monitoring applications (Tecchio et al., 2020b). In this study, we have transitioned from focusing on ‘morphological similarity’ to investigating ‘resting-state similarity’ via NCD, which directly examines local neurodynamics.

Recent findings show that as right-hand dominance increases, the similarity in the homologous corticospinal tracts’ recruitment patterns also increases (Pagliara et al., 2023). Furthermore, a neuromodulation intervention relieving chronic fatigue normalized these inter-lateral balances (Bertoli et al., 2023). This underscores the potential of using resting-state indices to evaluate hemispheric homology, particularly for chronic symptom management.

NCD builds upon its theoretical predecessor, the normalized information distance (NID), which relies on the Kolmogorov complexity of sequences. While potential optimality of NID is promising, its non-computability limits its practical use. In contrast, NCD, using real-world compressors, instead of Kolmogorov complexity, has shown remarkable versatility across various fields such as genomics, virology, linguistics, literature, music, and even plagiarism detection (Li and Vitányi, 1997; Li et al., 2004; Cilibrasi and Vitányi, 2005). In selecting NCD to compare neurodynamics across brain regions, we leveraged its ability to capture signal patterns, akin to the connectivity correlate of measures like Higuchi’s fractal dimension for local neurodynamics (Zappasodi et al., 2014, 2015; Smits et al., 2016; Porcaro et al., 2019; Olejarczyk et al., 2022). This sensitivity could be attributed to the recurrence of similar temporal patterns across different timescales, reflecting a common principle governing neuronal networks, whether at the level of single neurons, neuronal groups, or larger areas (Tecchio et al., 2020a; Persichilli et al., 2022). This principle, termed feedback-synchrony-plasticity (FeeSyCy), connects the individual with their environment in a purpose-dependent manner (Friston, 2010; Grujic et al., 2022).

Our protocols, including resting state and passive median nerve stimulation analyses enhanced by FSS (Barbati et al., 2006; Porcaro et al., 2008), are readily implementable in clinical settings, laying the groundwork for future studies on age- and pathology-related changes, especially in cases of hemispheric impairment like monolateral stroke. FSS analysis can evaluate resting-state power properties with minimal impact from generator position changes due to atrophy, particularly relevant in age-related contexts. This approach is feasible for most patients and is particularly beneficial for those with sensorimotor system pathologies, as FSS effectively identifies functionally homologous areas, even when displaced due to plastic reorganizations post-stroke (Tecchio et al., 2007; Rossini and Tecchio, 2008).

Interestingly, our findings revealed a pattern of hemispheric dominance, with the neurodynamics of left-dominant S1 showing more similarity across individuals than the right non-dominant. This counterintuitive result suggests that the neuronal activities of more skilled cortical areas become more similar within the population, possibly reaching a plateau of cortical functional potential.

NCD, by measuring signal similarity through compression lengths, introduces a novel approach to estimate functional brain connectivity directly from time-domain data, potentially preserving inherent patterns of local neurodynamics. Although the similarity of different regions in the same person has not been tested, the observation that homologous regions produce similar neurodynamics is in line with the concept that the neurodynamics of a certain brain region can serve as a “signature” of the generating region (Cottone et al., 2017; Armonaite et al., 2021, 2022). Given the critical role of homologous region balance in brain plasticity and learning, our approach could help in advancements our understanding chronic symptoms arising from imbalances in brain activity, such as depression, addiction, fatigue, and epilepsy, by taking advantage of the fact that the resting state offers a direct window into the chronic alterations underlying various behavioral dysfunctions.

## Data availability statement

The raw data supporting the conclusions of this article will be made available by the authors, without undue reservation.

## Ethics statement

The studies involving humans were approved by the Ethics Committee of S. Giovanni Calibita Hospital, Fatebenefratelli Isola Tiberina of Rome. The studies were conducted in accordance with the local legislation and institutional requirements. The participants provided their written informed consent to participate in this study.

## Author contributions

AP: Data curation, Formal analysis, Investigation, Supervision, Visualization, Writing – original draft. VB: Data curation, Formal analysis, Writing – review & editing. KA: Visualization, Writing – review & editing. CP: Supervision, Visualization, Writing – review & editing. LC: Supervision, Writing – review & editing. FC: Data curation, Writing – review & editing. LP: Data curation, Writing – review & editing, Conceptualization. DV: Visualization, Writing – review & editing. FT: Conceptualization, Data curation, Funding acquisition, Investigation, Methodology, Project administration, Resources, Software, Supervision, Validation, Visualization, Writing – original draft, Writing – review & editing.

## Appendix 1. FSS algorithm for S1 neuronal pools’ identification

FSS uses the information contained in recorded signal waveforms within a model suitable for electromagnetic signals, which in any time and spatial point are the sum of the signals generated by each ‘near-enough’ source.

FSS assumes the following model

$$x\left(t\right)=As\left(t\right)$$

where *x(t)* is the *m*-dimensional vector at time point *t* recorded by *m* MEG channels during the electrical stimulation of the contralateral median nerve at the wrist (*m* = 27 in our case); *A* is an *men* unknown mixing matrix and *s(t)* the *n*-dimensional unknown vector. In this respect, the model is the same of the Independent Component Analysis (ICA) one.

FSS estimates the Functional Source (FS) *y(t)* as

$$y\left(t\right)=Wx\left(t\right)$$

where *y(t)* is an *n*-dimensional vector, *W* is an *nxm* unmixing matrix that is estimated along with the independent components *y(t)*.

The aim of FSS is to enhance the separation of relevant signals by exploiting some a priori knowledge without renouncing the advantages of using only information contained in original signal waveforms.

FSS performs the following steps to obtain the activity of the primary somatosensory source (FS_S1) in the resting state:

1. Uses a contrast function modified with respect to standard ICA defined as

$$F=J+\mathrm{lambda}\;R\left(FS:S1\right)\text{,}$$

where *J* is the kurtosis used in fastICA, *lambda* is a parameter used to weigh the two parts of the contrast function and *R(FS_S1)* accounts for the prior information used to extract the single source *FS_S1* and is given by:

$$R\left(FS:S1\right)=\overset{t_{20}+\Delta_{2}t_{20}}{\underset{t_{20}-\Delta_{1}t_{20}}{\sum }}\left|EA\left(FS:S1,t\right)\right|-\overset{15}{\underset{10}{\sum }}\left|EA\left(FS:S1,t\right)\right|$$

where *EA* is the evoked activity computed by averaging signal epochs of the source *FS_S1* triggered on the median nerve stimulus at the wrist (*t* = 0); t_20_ is the time point with the maximum magnetic field value on the maximal original MEG channel around 20 ms after the stimulus arrival (searched in the [16–24] ms window); Δ_1_t_20_ (Δ_2_t_20_) is the time point corresponding to a field amplitude of 50 % of the maximal value, by definition in t_20_, before (after) t__20__; the baseline was computed in the no response time interval from 10 to 15 ms;

1. Finds the source *FS_S1* which globally maximizes the contrast function *F* by simulated annealing;
2. Computes the inverse of the estimated mixing vector A_*FS_S1*_ for the source *FS_S1*, up to permutation and scaling, as

$$W_{FS:S1}=1/A_{FS:S1}$$

1. Applies a semi-automatic artifact rejection procedure to the MEG data at rest to minimize the contribution of non-cerebral sources (such as the heart, eyes, muscles);
2. Multiplies the artifact-free rest MEG data *x(t)* by W_FS_S1_ to obtain *FS_S1* activity in the resting state

$$FS:S1\left(t\right)=W_{FS:S1}\;x\left(t\right)\text{.}$$

## Appendix 2. The concept of compressor in information theory

Let *X* be a given signal that takes values in a given set χ. It is then possible to estimate its empirical probability density function (i.e. its histogram) *p(x) = Pr{X=x}* with *x* ∈ *χ*. *X*‘s probability density function allows us to define the entropy of *X* as:

$$H\left(X\right)=-\sum x\in \chi \;p\left(x\right)\;\log\left(p\left(x\right)\right)\text{.}$$

*H(X)* indicates the ‘complexity’ of the signal *X*, which corresponds to how much we can compress it. More specifically, we can compress any signal *X* by well-known algorithms like *Huffman coding*, arithmetic coding etc. They are lossless coders, as they establish a bidirectional one-to-one correspondence between *X* and the compressed code. Typically, they exploit the simple principle of using a new code for each symbol *x* ∈ *χ*, where smaller length codes correspond to very frequent *X* and higher length codes correspond to not-frequent *X* of χ. The final effect is that the resulting coded signal has a number of bits *b* lower than the number of bits *B* of the original signal *X*, even though they contain the same information. The first *Shannon theorem* in Information Theory states that entropy *H* is the lowest number of bits per sample (of *X*) of any lossless code.

## References

[ref1] Armonaite K. Bertoli M. Paulon L. Gianni E. Balsi M. Conti L.. (2021). Neuronal electrical ongoing activity as cortical areas signature: an insight from MNI intracerebral recording atlas. Cereb. Cortex 32:2895:2906. doi: 10.1093/cercor/bhab389 34727186

[ref2] Armonaite K. Nobili L. Paulon L. Balsi M. Conti L. Tecchio F.. (2022). Local neurodynamics as a signature of cortical areas: new insights from sleep. Cereb. Cortex 33, 3284–3292. doi: 10.1093/cercor/bhac274, PMID: 35858209

[ref3] Barbati G. Porcaro C. Zappasodi F. Rossini P. M. Tecchio F. (2004). Optimization of an independent component analysis approach for artifact identification and removal in magnetoencephalographic signals. Clin. Neurophysiol. 115, 1220–1232. doi: 10.1016/j.clinph.2003.12.015, PMID: 15066548

[ref4] Barbati G. Sigismondi R. Zappasodi F. Porcaro C. Graziadio S. Valente G.. (2006). Functional source separation from magnetoencephalographic signals. Hum. Brain Mapp. 27, 925–934. doi: 10.1002/hbm.20232, PMID: 16575833 PMC6871330

[ref5] Bello J. P. (2011). Measuring structural similarity in music. IEEE Trans. Audio Speech Lang. Process. 19, 2013–2025. doi: 10.1109/TASL.2011.2108287

[ref6] Bertoli M. Tataranni A. Porziani S. Pasqualetti P. Gianni E. Grifoni J.. (2023). Effects on corticospinal tract homology of Faremus personalized neuromodulation relieving fatigue in multiple sclerosis: a proof-of-concept study. Brain Sci. 13:574. doi: 10.3390/BRAINSCI13040574 37190539 PMC10136421

[ref7] Carson R. G. (2020). Inter-hemispheric inhibition sculpts the output of neural circuits by co-opting the two cerebral hemispheres. J Physiol 598:4781:4802. doi: 10.1113/JP279793, PMID: 32770748

[ref8] Cataltepe Z. Yaslan Y. Sonmez A. (2007). Music genre classification using MIDI and audio features. EURASIP J. Adv. Signal Process. 2007:36409. doi: 10.1155/2007/36409

[ref9] Cilibrasi R. Vitányi P. M. B. (2005). Clustering by compression. IEEE Trans. Inf. Theory 51, 1523–1545. doi: 10.1109/TIT.2005.844059

[ref10] Cogliati Dezza I. Zito G. Tomasevic L. Filippi M. M. Ghazaryan A. Porcaro C.. (2015). Functional and structural balances of homologous sensorimotor regions in multiple sclerosis fatigue. J. Neurol. 262, 614–622. doi: 10.1007/s00415-014-7590-6, PMID: 25522694

[ref11] Cottone C. Porcaro C. Cancelli A. Olejarczyk E. Salustri C. Tecchio F. (2017). Neuronal electrical ongoing activity as a signature of cortical areas. Brain Struct. Funct. 222, 2115–2126. doi: 10.1007/S00429-016-1328-4, PMID: 27803994

[ref12] Cottone C. Tomasevic L. Porcaro C. Filligoi G. Tecchio F. (2013). Physiological aging impacts the hemispheric balances of resting state primary somatosensory activities. Brain Topogr. 26, 186–199. doi: 10.1007/S10548-012-0240-3, PMID: 22760422

[ref13] Das A. Gilbert C. D. (1999). Topography of contextual modulations mediated by short-range interactions in primary visual cortex. Nature 399, 655–661. doi: 10.1038/21371, PMID: 10385116

[ref14] Deco G. Corbetta M. (2011). The dynamical balance of the brain at rest. Neuroscientist 17, 107–123. doi: 10.1177/1073858409354384, PMID: 21196530 PMC4139497

[ref15] Dell’Acqua M. L. Landi D. Zito G. Zappasodi F. Lupoi D. Rossini P. M.. (2010). Thalamocortical sensorimotor circuit in multiple sclerosis: an integrated structural and electrophysiological assessment. Hum. Brain Mapp. 31, 1588–1600. doi: 10.1002/hbm.20961, PMID: 20162580 PMC6871076

[ref16] Friston K. (2010). The free-energy principle: a unified brain theory? Nat. Rev. Neurosci. 11, 127–138. doi: 10.1038/nrn2787 20068583

[ref17] Georgopoulos A. P. Carpenter A. F. (2015). Coding of movements in the motor cortex. Curr. Opin. Neurobiol. 33, 34–39. doi: 10.1016/j.conb.2015.01.012 25646932

[ref18] Gomez F. J. (2009). *Sustaining diversity using behavioral information distance*. In: Proceedings of the 11th annual genetic and evolutionary computation conference, GECCO-2009, pp. 113–120.

[ref19] Graziadio S. Basu A. Tomasevic L. Zappasodi F. Tecchio F. Eyre J. A. (2010). Developmental tuning and decay in senescence of oscillations linking the corticospinal system. J. Neurosci. 30, 3663–3674. doi: 10.1523/JNEUROSCI.5621-09.2010, PMID: 20220000 PMC6632255

[ref20] Graziadio S. Tomasevic L. Assenza G. Tecchio F. Eyre J. A. (2012). The myth of the “unaffected” side after unilateral stroke: is reorganisation of the non-infarcted corticospinal system to re-establish balance the price for recovery? Exp. Neurol. 238, 168–175. doi: 10.1016/j.expneurol.2012.08.031 22981842 PMC3508413

[ref21] Grujic N. Brus J. Burdakov D. Polania R. (2022). Rational inattention in mice. Sci. Adv. 8:eabj8935. doi: 10.1126/sciadv.abj8935, PMID: 35245128 PMC8896787

[ref22] Kolasinski J. Logan J. P. Hinson E. L. Makin T. R. Emir U. E. Stagg Correspondence C. J. (2017). A mechanistic link from GABA to cortical architecture and perception. Curr. Biol. 27, 1685–1691.e3. doi: 10.1016/j.cub.2017.04.055, PMID: 28552355 PMC5462622

[ref23] Li M. Chen X. Li X. Ma B. Vitányi P. M. B. (2004). The similarity metric. IEEE Trans. Inf. Theory 50, 3250–3264. doi: 10.1109/TIT.2004.838101

[ref24] Li M. Vitányi P. M. B. (1997). No title. New York: Springer.

[ref25] Mahan M. Y. Georgopoulos A. P. (2013). Motor directional tuning across brain areas: directional resonance and the role of inhibition for directional accuracy. Front. Neural Circuits 7:92. doi: 10.3389/fncir.2013.00092, PMID: 23720612 PMC3654201

[ref26] Nykter M. Price N. D. Aldana M. Ramsey S. A. Kauffman S. A. Hood L. E.. (2008). Gene expression dynamics in the macrophage exhibit criticality. Proc. Natl. Acad. Sci. U. S. A. 105, 1897–1900. doi: 10.1073/pnas.0711525105, PMID: 18250330 PMC2538855

[ref27] Olejarczyk E. Zappasodi F. Ricci L. Pascarella A. Pellegrino G. Paulon L.. (2022). Functional source separation-identified epileptic network: analysis pipeline. Brain Sci. 12:1179. doi: 10.3390/brainsci12091179, PMID: 36138915 PMC9496980

[ref28] Oliviero A. Tecchio F. Zappasodi F. Pasqualetti P. Salustri C. Lupoi D.. (2004). Brain sensorimotor hand area functionality in acute stroke: insights from magnetoencephalography. NeuroImage 23, 542–550. doi: 10.1016/j.neuroimage.2004.06.040, PMID: 15488403

[ref29] Ondobaka S. De Doncker W. Ward N. Kuppuswamy A. (2022). Neural effective connectivity explains subjective fatigue in stroke. Brain 145, 285–294. doi: 10.1093/brain/awab287, PMID: 34791073 PMC8967104

[ref30] Pagliara M. R. Cecconi F. Pasqualetti P. Bertoli M. Armonaite K. Gianni E.. (2023). On the homology of the dominant and non-dominant corticospinal tracts: a novel neurophysiological assessment. Brain Sci. 13:278. doi: 10.3390/brainsci13020278 36831821 PMC9954672

[ref31] Pascarella A. Gianni E. Abbondanza M. Armonaite K. Pitolli F. Bertoli M.. (2022). Normalized compression distance to measure cortico-muscular synchronization. Front. Neurosci. 16:933391. doi: 10.3389/fnins.2022.933391, PMID: 36440261 PMC9687393

[ref32] Pellegrino G. Tomasevic L. Tombini M. Assenza G. Bravi M. Sterzi S.. (2012). Inter-hemispheric coupling changes associate with motor improvements after robotic stroke rehabilitation. Restor. Neurol. Neurosci. 30, 497–510. doi: 10.3233/RNN-2012-120227, PMID: 22868224

[ref33] Persichilli G. Grifoni J. Pagani M. Bertoli M. Gianni E. L’Abbate T.. (2022). Sensorimotor interaction against trauma. Front. Neurosci. 16:913410. doi: 10.3389/fnins.2022.913410, PMID: 35774554 PMC9238294

[ref34] Porcaro C. Barbati G. Zappasodi F. Rossini P. M. Tecchio F. (2008). Hand sensory–motor cortical network assessed by functional source separation. Hum. Brain Mapp. 29, 70–81. doi: 10.1002/hbm.20367, PMID: 17318837 PMC6870883

[ref35] Porcaro C. Cottone C. Cancelli A. Rossini P. M. Zito G. Tecchio F. (2019). Cortical neurodynamics changes mediate the efficacy of a personalized neuromodulation against multiple sclerosis fatigue. Sci. Rep. 9:18213. doi: 10.1038/s41598-019-54595-z, PMID: 31796805 PMC6890667

[ref36] Rossini P. M. Tecchio F. (2008). On primary cortical hand representation in the left and right hemispheres. Clin. Neurophysiol. 119, 2421–2423. doi: 10.1016/j.clinph.2008.07.006 18789758

[ref37] Smits F. M. Porcaro C. Cottone C. Cancelli A. Rossini P. M. Tecchio F. (2016). Electroencephalographic fractal dimension in healthy ageing and Alzheimer’s disease. PLoS One 11:e0149587. doi: 10.1371/journal.pone.0149587, PMID: 26872349 PMC4752290

[ref38] Soleimani B. Dallasta I. Das P. Kulasingham J. P. Girgenti S. Simon J. Z.. (2023). Altered directional functional connectivity underlies post-stroke cognitive recovery. Brain Commun 5:149. doi: 10.1093/braincomms/fcad149, PMID: 37288315 PMC10243775

[ref39] Spetsieris P. G. Ko J. H. Tang C. C. Nazem A. Sako W. Peng S.. (2015). Metabolic resting-state brain networks in health and disease. Proc. Natl. Acad. Sci. U. S. A. 112, 2563–2568. doi: 10.1073/pnas.1411011112, PMID: 25675473 PMC4345616

[ref40] Tecchio F. Bertoli M. Gianni E. L’Abbate T. Paulon L. Zappasodi F. (2020a). To be is to become. Fractal Neurodynamics of the body-brain control system. Front. Physiol. 11:768. doi: 10.3389/fphys.2020.609768, PMID: 33384616 PMC7770125

[ref41] Tecchio F. Cecconi F. Colamartino E. Padalino M. Valci L. Reinert M. (2020b). The morphology of somatosensory evoked potentials during middle cerebral artery aneurysm clipping (MoSAC): a pilot study. Clin. EEG Neurosci. 51, 130–136. doi: 10.1177/1550059419874942, PMID: 31514539

[ref42] Tecchio F. Pasqualetti P. Pizzella V. Romani G. Rossini P. M. (2000). Morphology of somatosensory evoked fields: inter-hemispheric similarity as a parameter for physiological and pathological neural connectivity. Neurosci. Lett. 287, 203–206. doi: 10.1016/S0304-3940(00)01171-X, PMID: 10863030

[ref43] Tecchio F. Porcaro C. Barbati G. Zappasodi F. (2007). Functional source separation and hand cortical representation for a brain-computer interface feature extraction. J. Physiol. 580, 703–721. doi: 10.1113/jphysiol.2007.129163, PMID: 17331989 PMC2075454

[ref44] Tecchio F. Zappasodi F. Pasqualetti P. Rossini P. M. (2005). Neural connectivity in hand sensorimotor brain areas: an evaluation by evoked field morphology. Hum. Brain Mapp. 24, 99–108. doi: 10.1002/hbm.20073, PMID: 15468154 PMC6871686

[ref45] Tecchio F. Zappasodi F. Tombini M. Oliviero A. Pasqualetti P. Vernieri F.. (2006). Brain plasticity in recovery from stroke: an MEG assessment. Neuroimage 32, 1326–1334. doi: 10.1016/J.NEUROIMAGE.2006.05.004, PMID: 16806985

[ref46] Yoshizawa S. Terano T. Yoshikawa A. (2010). *Analyzing the effects of peer review activities in the EFL writings*. Proceedings of the 18th international conference on computers in education: Enhancing and sustaining new knowledge through the use of digital Technology in Education, ICCE 2010, pp. 738–742.

[ref47] Zappasodi F. Marzetti L. Olejarczyk E. Tecchio F. Pizzella V. (2015). Age-related changes in electroencephalographic signal complexity. PLoS One 10:e0141995. doi: 10.1371/journal.pone.0141995, PMID: 26536036 PMC4633126

[ref48] Zappasodi F. Olejarczyk E. Marzetti L. Assenza G. Pizzella V. Tecchio F. (2014). Fractal dimension of EEG activity senses neuronal impairment in acute stroke. PLoS One 9:e100199. doi: 10.1371/journal.pone.0100199, PMID: 24967904 PMC4072666

[ref49] Zatorre R. J. Fields R. D. Johansen-Berg H. (2012). Plasticity in gray and white: neuroimaging changes in brain structure during learning. Nat. Neurosci. 15, 528–536. doi: 10.1038/NN.3045, PMID: 22426254 PMC3660656

